# Effects of dietary humic acid and enzymes on meat quality and fatty acid profiles of broiler chickens fed canola-based diets

**DOI:** 10.5713/ajas.18.0408

**Published:** 2018-09-13

**Authors:** Amogelang R. P. Disetlhe, Upenyu Marume, Victor Mlambo, Arno Hugo

**Affiliations:** 1 Department of Animal Sciences, School of Agriculture Science, Faculty of Agriculture, Science and Technology, North West University, P Bag X 2046, Mmabatho 2735, South Africa; 2 Food Security and Safety Niche area, Faculty of Agriculture, Science and Technology, North West University, P Bag X 2046, Mmabatho 2735, South Africa; 3 Department of Microbial, Biochemical and Food Biotechnology, University of Free State, P.O. Box 339 Bloemfontein, 9300, South Africa

**Keywords:** Canola, Humic Acid, Enzymes, Meat Quality, Fatty Acids

## Abstract

**Objective:**

This study was conducted to assess the effect of potassium humate and enzymes (Xylanase+Amylase+Protease) inclusion in diets on carcass characteristics, meat quality and fatty acid profiles of broilers fed canola-based diets.

**Methods:**

Two hundred and twenty broilers randomly allotted to 5 dietary treatments: the control (commercial broiler diet); CM (17.5% canola meal inclusion); CMEnz (17.5% CM inclusion+0.3 g/kg Axtra XAP); CMPh (17.5% CM inclusion+1.5% Potassium Humate, PH); and CMEnzPh (17.5% CM inclusion+1.5% PH+0.3 g/kg Axtra XAP) were slaughtered at day 42 for assessment of carcass and meat quality parameters.

**Results:**

Diet had no effect on carcass traits apart from breast muscle weight and breast muscle index. The highest breast muscle weight was observed in broilers fed CMEnz (487.6±17.5 g) followed by those fed the control diet (474.37±17.5 g). Diet also had no significant dietary effect on pH, temperature, drip loss and shear force values of the breast muscle. However, diet significantly affected meat colour and water-holding capacity. Broilers in the control and CMPh groups (52.94±0.67 and 52.91±0.67) had the highest (p<0.05) values for lightness (L*), whilst those fed CMEnzPh had the lowest value (47.94±0.67). In contrast, CM group had the lowest (p<0.05) value for redness (a*) with CMEnzPh group having the highest values. The proportion of polyunsaturated fatty acids (PUFAs), n-6 and n-3 fatty acids and the PUFA/saturated fatty acid ratio were increased in CM-based diets containing enzymes and humic acid.

**Conclusion:**

It can, therefore, be concluded that CM can be included in broiler diets in the presence of enzymes and humic acid with positive effects on meat quality and important fatty acids that are beneficial to the health of consumers.

## INTRODUCTION

Meat and meat products with higher levels of minerals (iron, zinc, and selenium), polyunsaturated fatty acids (PUFAs) and antioxidants are attractive and valued worldwide [1]. Presently, the production and consumption of chicken meat and its products has significantly increased worldwide due to desirable nutritional characteristics. In comparison to beef and pork, chicken products contain high amounts of protein, low fat content and relatively high concentrations of PUFAs, which are beneficial to the health of consumers [2]. Generally, nutrition of the chicken plays a significant role in influencing the composition of meat. The reported health benefits PUFAs in meat products has created a trend towards increasing the content of these fatty acids, particularly the omega 3 PUFAs, in meat through dietary manipulation [1,3].

High value protein sources used in broiler diets, including soybean meal (SBM) are becoming expensive due to the competition between humans and livestock for food and feed. This has provoked intense efforts to explore alternative protein sources that include canola meal (CM). Canola has crude protein levels that range from 34% to 39% and has comparable amino acid profile to that of SBM. Its use in broiler diets can, however, be limited by high fibre content, which may influence digesta viscosity and decrease nutrient availability [4,5]. Inclusion of enzymes and organics acids such as humic acids have been observed to improve digestibility of poor protein sources such as CM. In several studies, digestibility, efficiency of feed utilisation and meat quality of broilers was improved when a xylanase, amylase and protease enzyme complex was added to the broiler diet [1,6–8]. More important, xylanase has been observed to enhance disruption of cell wall material in canola, thus releasing captured carbohydrates while amylase may increase the digestion of the released carbohydrates [4]. On the other hand, protease might break down a portion of protein that may escape digestion in the gut ultimately increasing protein digestion efficiency [4,9]. It can, therefore, be expected that the inclusion of different combinations of enzymes in broiler diets can be a strategy to improve the nutritional value of CM and, consequently, alter the composition of meat.

Information regarding the influence of humic acids, a natural feed additive, on the digestion and utilisation of poor protein sources such as CM in broilers is scarce. Humic acid is a natural organic acid and has been shown to influence digestion, immune response and general performance of broilers [10]. Its inclusion in broiler diets may stimulate changes in digestion dynamics, assimilation of nutrients and meat metabolite profiling, resulting in desirable meat compositional and organoleptic physiognomic quality [1]. In chicken and pork, humic acid inclusion in diets was observed to desirably modify meat colour mainly due to accelerated myoglobin synthesis [10–12]. Moreover, in pork, humic acid was observed to have an effect of increasing the fat marbling values and to reduce back fat thickness, probably due its influence on protein and lipid distribution [12]. To our knowledge, no study has been conducted to evaluate the influence of humic acid on meat quality and fatty acid profiles in broilers fed poor protein sources such as CM. Therefore, the current study was designed to determine the effects of dietary humic acid and enzymes on carcass characteristics, meat quality and fatty acid parameters in broilers fed canola-based diets.

## MATERIALS AND METHODS

### Experimental design and diets

A total of two hundred and twenty birds (Cobb 500) from a feeding trial were used in the study. All chickens were given broiler starter mash from day 1 to 14. At the beginning of the grower phase (d 15), the birds were randomly allotted to 5 dietary treatments replicated 4 times with a pen housing 11 birds as the experimental unit. The study was arranged in a completely randomized design. The pens (measuring 3.5×1.0×1.85 m) were designed to meet the animal welfare standards for optimum production of broilers. Five dietary treatments in form of mash were formulated with the control diet being composed of a commercial grower diet whose major protein source was SBM, whilst the other four diets contained CM at 17.5% inclusion in place of SBM. The dietary treatments were formulated as follows: i) Control (commercial broiler diet); ii) CM (17.5% canola meal inclusion); iii) CMEnz (CM+0.3 g/kg Axtra XAP); iv) CMPh (CM+1.5% Potassium Humate, PH); v) CMEnzPh (CM+1.5% PH+0.3g/kg Axtra XAP). The 17.5% Canola inclusion was the maximum rate allowable for formulation of a balanced broiler diet, whilst the inclusion level of enzymes (Xylanase, Amylase, and Protease [g], Axtra XAP, OPTIFEEDS, Lichtenburg, South Africa) was the recommended level provided by feed companies for ingredients such as Canola, which are poor protein sources containing high fibre. Humic acid was added in the form of a humic acid salt (potassium humate, PH, NUTRICO, Johannesburg, South Africa). The inclusion level of humic acid was derived based on the ranges from literature [10,13].

Ingredients and dietary formulations are shown in Table 1 whilst Table 2 shows the nutritional compositions of the diets. Near-infrared reflectance spectroscopy was used to determine the proximate composition of the diets. The experimental diets were only offered during the grower (d 15 to 28) and finisher (d 29 to 42) phases. At the end of the feeding trial (d 42), all broilers were deprived of feed for a period of 13 hours to allow the clearing of the crop and subsequently slaughtered for assessment of carcass traits, meat quality and fatty acid profiles. All the study procedures were conducted at the North-West University experimental farm. The experimental procedures were approved by the Animal Research Ethics Committee of North-West University and the Ethics number granted is NWU-00516-16-S9.

### Carcass traits and internal organs

At slaughter, the birds were electrically stunned, exsanguinated, defeathered and eviscerated in accordance with the standard procedures for humane animal slaughter. The carcasses were weighed and packed in labelled plastics bags for each treatment. The internal organs (including the gizzards, livers, hearts, spleens, and intestines) were removed and packaged separately in labelled plastics bags and weighed individually. Lengths of small intestines (jejunum, duodenum, and ileum) were also measured individually and recorded. The hot carcass weight of each chicken was recorded and dressing out percentage was calculated. The carcasses were then chilled overnight, reweighed to obtain the cold carcass weight and thereafter the breast and thigh samples were obtained and weighed to determine the breast muscle and thigh muscle weights and ratios. Breast muscles were also obtained for the evaluation of meat quality traits and fatty acid profiles.

### Meat quality measurement

Meat pH (pH_u_) and temperature measurements were taken 24 hours post-slaughter on the breast muscle (central area of the breast) using a Corning Model 4 pH-temperature meter (Corning Glass Works, Medfield, MA, USA). Colour of the meat (ΔL* = lightness, Δa* = redness, and Δb* = yellowness) was measured using a Minolta colour-guide (Spectrophotometer CM 2500c, Konika Minolta, Osaka, Japan). The water holding capacity (WHC) was determined as the amount of water expressed from fresh meat held under pressure (60 kg pressure) using the filter-paper press. Drip loss was determined as the difference in weight of meat sample suspended in bottle and stored in a cold room at 4°C for 72 hours before and after drip. The meat cooking loss was calculated as the difference between the weight of raw meat and cooked meat while meat tenderness was determined using a Meullenet - Owens Razor Shear Blade (A/MORS) mounted on a Texture analyser (TA XT plus, Stable Micro Systems, Surrey, UK). The reported value in Newtons (N) represented the average of the peak force measurements of each sample.

### Fatty acid analysis

Total lipids of feed (Table 3) and muscle samples were quantitatively extracted, according to the method of Folch et al [14] using chloroform and methanol in a ratio of 2:1, while fatty acid methyl esters (FAMEs) were quantified using Varian 430 flame ionization gas chromatography, with a fused silica capillary column, Chrompack CPSIL 88 (100 m length, 0.25 mm ID, 0.2 μm film thicknesses) according to the procedures described by Aldai et al [15] and Alfaia et al [16]. Standards for identification of the FAMEs were obtained from Sigma-Aldrich (Aston Manor, Pretoria, South Africa) while all other reagents and solvents were of analytical grade were attained from Merck Chemicals, (Halfway House, Johannesburg, South Africa). Individual fatty acids were calculated as a proportion of total fatty acids present in the sample, while total fatty acids combinations were calculated as: omega-3 (n-3) fatty acids, omega-6 (n-6) fatty acids, total saturated fatty acids (SFA), total monounsaturated fatty acids (MUFA), PUFA, PUFA/SFA ratio, and n-6/n-3 ratio.

### Statistical analysis

Data on carcass characteristics, meat quality parameters and fatty acid profiles of broiler meat were analysed using general linear model procedure of SAS [17]. The statistical model was as follows:

$${\text{Y}}_{ij}=\mu +{\text{T}}_{i}+{ɛ}_{ij}$$

Where: Y*_ij_* = observation (carcass characteristics, meat quality parameters and fatty acid profiles), μ = population mean constant common to all observations, T*_i_* = effect of diet, and ɛ*_ij_* = random error term. The probability differences (PDIFF) option of SAS [17] was used to perform pairwise comparisons and for all tests, the level of significance was set at (p< 0.05).

## RESULTS

### Carcass traits and internal organs

Results from the study showed a significant effect of diet on the breast muscle weight and breast muscle ratio (Table 4). The breast muscle weight and the ratio are indicative of muscle mass in a carcass. The highest breast muscle weight was observed in chickens offered CMEnz (487.6±17.5 g) diet followed by chickens fed CMPh (474.37±17.5 g). The CM fed broilers had the lowest breast weight. Similarly, CMEnz broilers had the highest breast muscle ratio followed by the CMPh. The CM fed broilers had the lowest breast muscle ratio. The weights of the internal organs (gizzard, heart, spleen) and intestinal length (duodenum, jejunum, ileum) were significantly (p<0.05) influenced by the diets (Table 5). The gizzard and spleen of broilers in the CMEnzPh treatment were heavier (p<0.05) than in broilers offered CMEnz. Chickens fed the CMEnzPh diet also had the longest jejunum followed by CMPh. The heart weight was heaviest (p<0.05) in CMEnz (25.90 g) whilst CMPh and CMEnzPh chickens had the lowest heart weights.

### Meat quality

The results on meat quality measurements showed that diet only affected meat colour and WHC (Table 6). The findings of the current study, show an increase in redness (Δa*) with the inclusion of enzymes in the diets. However, broilers fed the Control and CMPh diets had lighter (ΔL*) breast muscles. Meat of broilers fed the control diet had the lowest water loss, which means it has the greatest capacity to retain water, whilst the meat of broilers supplemented with CMEnz showed the least capacity to retain water. In the current study, the effect of diet on shear force values was insignificant.

### Fatty acid profiles and nutritional indices of broiler meat

Diet had no effect (p>0.05) on the intramuscular fat (IMF) (Table 7). In the current study, the predominant fatty acids included the palmitic acid (C16:0), oleic acid (C18:1), linoleic acid (C18:2 n-6), and stearic acid (Table 8). Broilers fed on the control diet had higher values for most of the SFA, particularly myristic (C14:0) and palmitic (C16:0) acids. However, the canola fed broilers had higher values for most PUFAs including, the linoleic (C18:2 n-6), γLinolenic (C18:3 n-3), eicosadienoic, eicosatrienoic, decosapentaenoic, and decosahexaenoic acids. Interestingly, inclusion of humic acid in diets appeared to significantly increase the proportions of the n-3 PUFAs in broiler meat. The meat of broilers fed canola based diets were observed to be rich in PUFAs, total n-6 fatty acids and total n-3 fatty acids (Table 9). In the current study, inclusion of CM, humic acid and enzymes in diets resulted in an increase in PUFA/SFA ratio. Moreover, the inclusion of canola, humic acid and enzymes also reduced the n-6/n-3 ratio, particularly in the CMPh fed broilers although the values were above the recommended ratio of 5.

## DISCUSSION

### Carcass traits and internal organs

The higher breast weights of broilers fed humic acid and enzyme complex diets could be attributed to the effect of the humic acid and an enzyme complex in stimulating effective digestion and muscle accretion. According to Li et al [18] the inclusion of humic acid in broiler diets can improve digestion dynamics and nutrient absorption ultimately regulating growth and change the metabolism to enhance animal carcass traits. Moreover, Ozturk et al [10] and Kocabagli et al [19] also demonstrated a linear increase in body and carcass weights with the inclusion of humic acid substances in the broiler diet. On the other hand Dalólio et al [20], also reported a significant improvement on breast performance at 42 days for broilers supplemented with enzyme complex as observed in the current study. Diet, including other factors such as genetics, sex, slaughtering conditions and age of the animal can influence carcass traits [21,22]. Contrary to the current study Mateos et al [23] and Young et al [24] reported that broiler breast yield was reduced significantly when a diet of SBM was replaced with canola meal. Observation from the current study therefore confirm that inclusion of enzymes can improve utilisation of CM in broiler diets. The observed lack of differences in all other traits, apart from breast muscle weight and breast muscle ratio, in the current study is consistent with findings reported by Gopinger et al [25] where there was no effect of diet on carcass characteristics on broilers fed different protein sources at the growth and finisher phases.

Changes in size and structure of internal organs can be indicative of the effect of diet and its components on the development and function of the organs. The observed increase in weights of gizzards and spleen, and intestinal length could be attributed largely to the trophic effect of humic acid in stimulating the proliferation of normal cells and tissues, enhancing healthy tissue turnover and maintenance [18,26]. Moreover, the intestinal morphological alterations enhanced by humic acid could have the effect of increasing retention time of feed for digestion processes and enhancing mucosal permeability for efficient nutrient assimilation [26–28]. Findings from other studies, however, revealed lack of effect of diet on internal organs [29,30].

### Meat quality

The visual appearance of meat is one of the most vital meat quality attributes that influence acceptance of meat and meat products and purchasing decisions by consumers [25,31]. The findings of the current study, showing lighter breast muscles in broilers fed the Control and CMPh diets are consistent with findings by Wang et al [12] and Adeyemi and Sazili [32] who observed that humic acid inclusion in diets can improve the appearance of the meat. Although the precise underlying mechanism of the effect of humic substances is unknown, it appears that humic substances contains minute quantities of several minerals, including Fe, Mn, and Cu, which may influence meat colour [33]. The observation that meat of broilers fed the control diet had the greatest WHC is in concordance with findings from other studies [34–36]. Although diet had no effect on both drip loss and cooking loss, it has been observed that, generally, greater drip loss may induce a reduction in water-holding capacity and tenderness of meat [12,37]. The WHC is generally associated with the lipid peroxides content in the muscle [38,39]. In the current study, the effect of diet on shear force values was insignificant. The shear force is an objective indicator of the toughness of the meat [40–42]. A lower shear force value is indicative of meat that is tenderer [25,42].

### Fatty acid profiles and nutritional indices of broiler meat

Generally intra-muscular fat (IMF-marbling) content is an intrinsic indicator of meat quality [7,43]. The observed lack of influence of diet on IMF in the current study suggests that inclusion of CM, enzyme complex and humic acid salt in diets had no influence on fat biosynthesis during the regulation of IMF content [44]. Nevertheless the observed predominant fatty acids included the palmitic acid (C16:0), oleic acid (C18:1), linoleic acid (C18:2 n-6) and stearic acid are consistent with findings from other studies [3,45]. Broilers fed on the control diet had higher values for most of the SFA, particularly myristic (C14:0) and palmitic (C16:0) acids, which have a great significance due to their hypercholesterolemic properties that are related to coronary heart disease [3,46]. However, the canola fed broilers had higher values for most PUFAs including, the linoleic (C18:2 n-6), γLinolenic (C18:3 n-3), eicosadienoic, eicosatrienoic, decosapentaenoic, and decosahexaenoic acids which are essential for human health. Interestingly, inclusion of humic acid in diets appeared to significantly increase the proportions of the n-3 PUFAs in broiler meat. Although the underlying mechanism is still not well understood, in pork, humic acid was observed to increase fat marbling values and to reduce back fat thickness probably due its influence on protein and lipid digestibility and distribution that consequent in increased PUFA storage in the muscle tissue [12]. The increase in proportion of n-3 PUFAs in the CMPh fed broilers could also have been due to the influence of humic acid in increasing the efficiency of desaturase activities in the conversion of γLinolenic (ALA C18:3 n-3) to eicosapentaenoic acid (EPA 20:5n-3), docosapentaenoic (DPA 20:5 n-3), and docosahexaenoic acid (DHA 22:6n-3) [12].

The high PUFAs, total n-6 fatty acids and total n-3 fatty acids concentrations observed in broilers fed canola based diets could be a result of the contribution of protein source that was used in the diets in synergy with the activities of the feed additives in modulating the synthesis of intrinsic beneficial fatty acids that promotes the health of consumers. This is also supported by the higher values of PUFAs, total n-6 fatty acids and total n-3 fatty acids observed in the Canola-based diets (Table 3). According to FAO/WHO [47] and Grashorn [48], both n-3 and n-6 FAs in the meat play an immense role in human nutrition as they are originators of eicosanoids, leucotriens, and thromboxanes, which regulate the cardiovascular system and immunological processes [3]. The PUFA:SFA and n-6/n-3 ratios are critical parameters used to evaluate the nutritional value of meat [3,49]. Generally, meat with low PUFA:SFA ratio which is less than (0.4) and high n-6/n-3 ratio (>5) ratio may be considered to be poor and unfavourable since they might encourage an escalation in cholesterolaemia [49]. In the current study, inclusion of CM, humic acid and enzymes in diets resulted in an increase in PUFA/SFA ratio. Moreover, the inclusion of canola, humic acid and enzymes also reduced the n-6/n-3 ratio, particularly in the CMPh fed broilers although the values were above the recommended ratio of 5. Therefore, inclusion of canola, humic acid and enzymes resulted in a favourable effect on the level of important FAs required for maintenance of human health.

## CONCLUSION AND APPLICATIONS

From the results, it can be concluded that the inclusion of enzymes (Axtra XAP) and potassium humate in canola-based broiler diets had beneficial effects on the carcass and meat quality parameters in terms of breast weights, WHC and colour coordinates. Inclusion of enzymes and potassium humate in canola-based broiler diets increased the proportion of PUFAs, n-6 and n-3 fatty acids and the PUFA/SFA ratio, which are important indicators of nutritional value of meat. Generally, inclusion of potassium humate in CM based diets alone resulted in greater benefits on intrinsic meat quality properties of broilers than that of the enzyme and the combination of the two in most instances did not yield any additional benefits.

## Figures and Tables

**Table 1:** Ingredients composition of experimental diets for grower and finisher broilers

Ingredients	Dietary treatments1)									

	Grower					Finisher				
	
	Control	CM	CMEnz	CMPh	CMEnzPh	Control	CM	CMEnz	CMPh	CMEnzPh
Yellow maize-fine	69.90	69.50	69.50	69.50	69.50	76.20	76.40	76.40	76.40	76.4
Canola oilcake (HEX)	0.00	17.50	17.50	17.50	17.50	0.00	17.50	17.50	17.50	17.50
Prime gluten 60 (Yellow)	1.80	2.40	2.40	2.40	2.40	1.27	1.80	1.80	1.80	1.80
Fullfat soya	5.10	5.10	5.10	5.10	5.10	1.53	1.61	1.60	1.59	1.54
Soybean meal (Local)	19.70	2.22	2.21	2.22	2.22	18.00	0.50	0.60	0.50	0.50
Limestone powder-fine	1.45	1.22	1.22	1.22	1.22	1.30	1.07	1.07	1.07	1.07
MCP/Mono Cal KK	0.72	0.56	0.56	0.56	0.56	0.50	0.33	0.33	0.33	0.33
Salt-fine	0.32	0.32	0.32	0.32	0.32	0.33	0.33	0.33	0.33	0.33
Bicarbonate	0.17	0.16	0.16	0.16	0.16	0.13	0.12	0.12	0.12	0.12
Choline powder	0.08	0.08	0.08	0.08	0.08	0.08	0.08	0.08	0.08	0.08
Lysine	0.28	0.29	0.29	0.29	0.29	0.26	0.27	0.27	0.27	0.27
L-threonine	0.04	0.00	0.00	0.00	0.00	0.03	0.00	0.00	0.00	0.00
Methionine	0.19	0.18	0.18	0.18	0.18	0.16	0.09	0.09	0.09	0.09
PX P2 Br Gr with Phytase	0.17	0.17	0.17	0.17	0.17	0.00	0.00	0.00	0.00	0.00
PX P3 Br Fin with Phytase	0.00	0.00	0.00	0.00	0.00	0.17	0.17	0.17	0.17	0.17
Coxistac	0.05	0.05	0.05	0.05	0.05	0.05	0.05	0.05	0.05	0.05
Olaquindox	0.04	0.04	0.04	0.04	0.04	0.04	0.04	0.04	0.04	0.04
Axtra XAP (g/kg)	0.00	0.00	0.30	0.00	0.30	0.00	0.00	0.30	0.00	0.30
Potassium humate (%)	0.00	0.00	0.00	1.50	1.50	0.00	0.00	0.00	1.50	1.50

1) Dietary treatments: Control, commercial broiler diet; CM, commercial broiler diet in which 17.5% of soybean meal was replaced by canola meal; CMEnz, CM diet+0.3 g/kg Axtra XAP enzyme complex; CMPh, CM diet+1.5% potassium humate; and CMEnzPh, CM diet+1.5% PH+0.3 g/kg Axtra XAP enzyme complex.

**Table 2:** Nutrient composition (%) of experimental diets for grower and finisher broilers

Parameters	Standard broiler diet composition		Canola oil cake diet composition	
	
	Grower	Finisher	Grower	Finisher
Volume	100	100	100	100
Moisture	10.93	11.27	11.34	11.57
Metabolizable energy (MJ/kg)	11.79	11.9	12.09	12.29
Protein	18.93	16.89	18.80	16.90
Fat	6.24	6.38	5.96	5.99
Fibre	4.17	4.02	4.31	4.20
Ash	4.84	4.25	4.81	4.21
Linoleic	2.97	2.52	2.96	2.55
Choline (mg/kg)	1,285	1,159	1,283	1,173
Calcium	0.85	0.75	0.85	0.75
Phosphorus	0.56	0.50	0.59	0.49
Sodium	0.18	0.16	0.17	0.17
Chlorine	0.3	0.29	0.29	0.3
Potassium	0.73	0.65	0.76	0.68
Arginine	1.10	0.95	1.10	0.95

**Table 3:** Total fatty acid composition (%) of the commercial and canola-based diets

Parameter	Dietary treatments1)					SEM

	Control	CM	CMEnz	CMPh	CMEnzPh	
Total SFA	20.57^a^	15.28^b^	14.91^b^	15.1^b^	15.1^b^	0.14
Total MUFA	31.67^b^	32.06^a^	32.01^a^	31.75^ab^	31.76^ab^	0.10
Total PUFA	47.76^b^	52.66^a^	53.07^a^	53.17^a^	53.16^a^	0.23
Total n-6	46.06^b^	48.29^a^	48.52^a^	48.72^a^	48.69^a^	0.16
Total n-3	1.72^b^	4.37^b^	4.55^a^	4.45^a^	4.48^a^	0.21
PUFA:SFA	2.33^b^	3.45^a^	3.56^a^	3.52^a^	3.53^a^	0.13
n-6/n-3	26.77^a^	11.07^b^	10.67^b^	10.96^b^	10.88^b^	0.10

SEM, standard error of the mean; SFA, total saturated fatty acids; MUFA, total mono unsaturated fatty acids; PUFA, total poly unsaturated fatty acids; n-6, total omega-6 fatty acids; n-3, total omega-3 fatty acids.

1) Dietary treatments: Control, commercial broiler diet; CM, 17.5% CM inclusion; CMEnz, 17.5% CM inclusion+0.3 g/kg Axtra XAP; CMPh, 17.5% CM inclusion+1.5% Potassium Humate; CMEnzPh, 17.5% CM inclusion+1.5% PH+0.3 g/kg Axtra XAP.

a,b,c Means in the same row with different superscripts are significantly different (p<0.05).

**Table 4:** The effect of potassium humate and enzyme inclusions on carcass characteristics and meat quality measurements of broilers fed canola-based diets

Parameters	Dietary treatments1)					SEM

	Control	CM	CMEnz	CMPh	CMEnzPh	
Final body weight (g)	2,585	2,507	2,574	2,605	2,630	74.94
Hot carcass weight (g)	1,806.25	1,870	1,915.25	1,970	1,963.75	106.19
Cold carcass weight (g)	1,753	1,799.25	1,893.50	1,899.75	1,889.25	46.09
Dressing %	69.92	69.02	72.05	75.52	74.64	4.42
Av Breast weight (g)	422.82^b^	383^c^	487.6^a^	474.37^a^	487^a^	17.5
Av Thigh weight (g)	264.10	254.06	277.61	269.79	277.06	7.48
Breast muscle ratio	0.22^b^	0.23^b^	0.27^a^	0.26^a^	0.26^a^	0.01
Thigh muscle ratio	0.15	0.15	0.15	0.14	0.15	0.01

SEM, standard error of the mean.

1) Dietary treatments: Control, commercial broiler diet; CM, 17.5% CM inclusion; CMEnz, 17.5% CM inclusion+0.3 g/kg Axtra XAP; CMPh, 17.5% CM inclusion+1.5% Potassium Humate; CMEnzPh, 17.5% CM inclusion+1.5% PH+0.3 g/kg Axtra XAP.

a,b Means in the same rows with different superscripts are significantly different (p<0.05).

**Table 5:** The effect of potassium humate and enzyme inclusions on internal organs of broilers fed canola-based diets

Parameters	Dietary treatments1)					SEM

	Control	CM	CMEnz	CMPh	CMEnzPh	
Gizzard (g)	30.75^c^	30.90^c^	27.70^d^	33.10^b^	35.15^a^	1.06
Heart (g)	12.6^b^	13.55^a^	12.6^b^	11.70^c^	11.52^c^	0.21
Spleen (g)	2.45^d^	2.35^d^	2.85^c^	4.15^b^	4.69^a^	0.18
Duodenum (cm)	38.54^a^	31.24^c^	36.36^b^	35.81^b^	36.20^b^	2.69
Jejunum (cm)	41.28^c^	35.28^d^	45.10^b^	44.80^b^	47.64^a^	4.87
Ileum (cm)	52.34^a^	41.70^b^	53.68^a^	42.74^b^	43.34^b^	3.66
Liver (g)	41.55	42.85	42.69	40.65	40.00	1.27
HIS	0.023	0.024	0.023	0.023	0.022	0.01

SEM, standard error of the mean; HIS, hepatosomatic index.

1) Dietary treatments: Control, commercial broiler diet; CM, 17.5% CM inclusion; CMEnz, 17.5% CM inclusion+0.3 g/kg Axtra XAP; CMPh, 17.5% CM inclusion+1.5% Potassium Humate; CMEnzPh, 17.5% CM inclusion+1.5% PH+0.3 g/kg Axtra XAP.

a,b Means in the same rows with different superscripts are significantly different (p<0.05).

**Table 6:** The effect of potassium humate and enzyme inclusions on meat quality measurements of broilers fed canola-based diets

Parameters	Dietary treatments1)					SEM

	Control	CM	CMEnz	CMPh	CMEnzPh	
Ultimate pH	6.12	6.16	6.12	5.95	5.89	0.09
Ultimate temperature (°C)	14.63	12.56	14.08	15.54	16.69	0.69
Meat colour						
ΔL*	52.94^a^	50.79^b^	51.35^ab^	52.94^a^	47.94^c^	0.67
Δa*	1.81^c^	1.59^d^	2.46^b^	1.81^c^	3.42^a^	0.41
Δb*	13.91^b^	13.33^b^	13.69^b^	14.30^a^	13.71^b^	0.69
Water holding capacity (%)	29.34^c^	33.62^b^	37.47^a^	34.97^b^	33.04^b^	1.93
Drip loss (%)	9.58	8.45	9.69	10.91	9.22	1.02
Cooking loss (%)	13.75	14.73	18.07	14.45	12.40	1.47
Shear force (N)	6.47	6.38	8.32	8.81	8.31	1.07

SEM, standard error of the mean; L*, lightness; a*, redness; b*, yellowness.

1) Dietary treatments: Control, commercial broiler diet; CM, 17.5% CM inclusion; CMEnz, 17.5% CM inclusion+0.3 g/kg Axtra XAP; CMPh, 17.5% CM inclusion +1.5% Potassium Humate; CMEnzPh, 17.5% CM inclusion+1.5% PH+0.3 g/kg Axtra XAP.

a,b Means in the same rows with different superscripts are significantly different (p<0.05).

**Table 7:** Effects of potassium humate and enzyme dietary inclusions on proximate fat composition (%) of breast muscle of broiler fed canola-based diets

Parameter	Dietary treatments1)					SEM

	Control	CM	CMEnz	CMPh	CMEnzPh	
IMF	1.67	1.73	1.50	1.31	1.54	0.16
FFDM	22.99^b^	22.87^a^	22.11^c^	24.18^a^	22.58^b^	0.27
Moisture	75.34^ab^	75.40^ab^	76.38^a^	74.51^b^	75.87^a^	0.24

SEM, standard error of the mean; IMF, intramuscular fat; FFDM, fat free dry matter.

1) Dietary treatments: Control, commercial broiler diet; CM, 17.5% CM inclusion; CMEnz, 17.5% CM inclusion+0.3 g/kg Axtra XAP; CMPh, 17.5% CM inclusion+1.5% Potassium Humate; CMEnzPh, 17.5% CM inclusion+1.5% PH+0.3 g/kg Axtra XAP.

a,b,c Means in the same row with different superscripts are significantly different (p<0.05).

**Table 8:** Effects of potassium humate and enzyme dietary inclusions on fatty acid composition (%) of breast muscle from broiler fed canola-based diets

Fatty acids	Dietary treatments1)					SEM

	Control	CM	CMEnz	CMPh	CMEnzPh	
Myristic (C14:0)	0.37^a^	0.29^b^	0.27^c^	0.29^b^	0.29^b^	0.01
Myristoleic (C14:1c9)	0.07^a^	0.03^b^	0.02^b^	0.00^b^	0.01^b^	0.01
Pentadecylic (C15:0)	0.08	0.08	0.07	0.09	0.08	0.01
Palmitic (C16:0)	23.86^a^	21.08^c^	21.18^bc^	22.07^b^	21.86^bc^	0.24
Palmitoleic (C16:1)	4.65^a^	2.58^b^	2.35^b^	1.80^c^	2.46^b^	0.24
Margaric (C17:0)	0.94	0.96	1.05	1.29	0.93	0.09
Heptadecenoic (C17:1)	0.06	0.14	0.19	0.09	0.14	0.07
Stearic acid (C18:0)	8.01^c^	8.09^c^	9.07^b^	9.84^a^	8.39^b^	0.42
Oleic (C18:1)	30.43^a^	26.72^b^	26.28^b^	25.36^c^	26.67^b^	0.77
Vaccenic (C18:1, 7)	4.54^a^	4.39^b^	4.39^b^	4.07^c^	4.27^b^	0.09
Nonoadecanoic (C19:0)	0.47	0.48	0.53	0.66	0.46	0.05
Linoleic (C18:2, n-6)	19.33^c^	25.54^a^	24.27^a^	22.98^b^	24.96^a^	0.65
Arachidic (C20:0)	0.04	0.05	0.04	0.04	0.03	0.01
α-Linolenic (C18:3 n-3)	1.02^d^	1.96^a^	1.76^b^	1.62^c^	1.79^b^	0.09
Heneicosanoic (C21:0)	0.24	0.25	0.27	0.33	0.23	0.03
Eicosadienoic (C20:2, n-6)	0.36^c^	0.67^a^	0.59^b^	0.67^a^	0.57^b^	0.57
Eicosatrienoic (C20:3 n-6)	0.03^c^	0.10^a^	0.09^b^	0.11^a^	0.07^b^	0.01
Erucic (C22:1)	0.83	0.76	0.75	0.84	0.70	0.07
Arachidonic (C20:4 n-6)	3.72^c^	4.39^b^	5.25^a^	5.94^a^	4.69^b^	0.49
Eicosopentaenoic C20:5 n-3)	0.20	0.22	0.19	0.26	0.20	0.03
Docosapentaenoic (C22:5 n-3)	0.32^c^	0.63^b^	0.69^b^	0.81^a^	0.62^b^	0.06
Docosahexanoic (C22:6 n-3)	0.18^c^	0.38^b^	0.46^b^	0.61^a^	0.37^b^	0.06

SEM, standard error of the mean.

1) Dietary treatments: Control, commercial broiler diet; CM, 17.5% CM inclusion; CMEnz, 17.5% CM inclusion +0.3 g/kg Axtra XAP; CMPh, 17.5% CM inclusion+1.5% Potassium Humate; CMEnzPh, 17.5% CM inclusion+1.5% PH+0.3 g/kg Axtra XAP.

a,b,c Means in the same row with different superscripts are significantly different (p<0.05).

**Table 9:** The effect of potassium humate and enzyme inclusions on total fatty acid profiles (%, total nutritional indices) of broiler meat

Parameter	Dietary treatments1)					SEM

	Control	CM	CMEnz	CMPh	CMEnzPh	
Total SFA	34.00^a^	31.28^c^	32.48^b^	34.58^a^	32.28^b^	0.68
Total MUFA	40.61^a^	34.61^b^	33.99^bc^	32.17^c^	34.25^b^	4.25
Total PUFA	25.39^c^	34.09^a^	33.53^b^	33.25^b^	33.47^b^	0.62
Total n-6	23.52^c^	30.68^a^	30.23^a^	29.73^b^	30.33^a^	0.58
Total n-3	1.88^c^	3.42^a^	3.29^ab^	3.52^a^	3.14^b^	0.16
PUFA:SFA	0.75^c^	1.09^a^	1.03^a^	0.96^b^	1.04^a^	0.12
n-6/n-3	12.56^a^	8.99^bc^	9.22^b^	8.47^c^	9.68^b^	0.21

SEM, standard error of the mean; SFA, total saturated fatty acids; MUFA, total mono unsaturated fatty acids; PUFA, total poly unsaturated fatty acids; n-6, total omega- 6 fatty acids; n-3, total omega-3 fatty acids.

1) Dietary treatments: Control, commercial broiler diet; CM, 17.5% CM inclusion; CMEnz, 17.5% CM inclusion +0.3 g/kg Axtra XAP; CMPh, 17.5% CM inclusion+1.5% Potassium Humate; CMEnzPh, 17.5% CM inclusion+1.5% PH+0.3 g/kg Axtra XAP.

a,b,c Means in the same row with different superscripts are significantly different (p<0.05).
